# Protein complexes predictions within protein interaction networks using genetic algorithms

**DOI:** 10.1186/s12859-016-1096-4

**Published:** 2016-07-25

**Authors:** Emad Ramadan, Ahmed Naef, Moataz Ahmed

**Affiliations:** Department of Information and Computer Science, King Fahd University of Petroleum and Minerals, Dhahran, Saudi Arabia

**Keywords:** Protein complex detection, Protein–protein interaction network, Genetic algorithms, Graph clustering

## Abstract

**Background:**

Protein–protein interaction networks are receiving increased attention due to their importance in understanding life at the cellular level. A major challenge in systems biology is to understand the modular structure of such biological networks. Although clustering techniques have been proposed for clustering protein–protein interaction networks, those techniques suffer from some drawbacks. The application of earlier clustering techniques to protein–protein interaction networks in order to predict protein complexes within the networks does not yield good results due to the small-world and power-law properties of these networks.

**Results:**

In this paper, we construct a new clustering algorithm for predicting protein complexes through the use of genetic algorithms. We design an objective function for exclusive clustering and overlapping clustering. We assess the quality of our proposed clustering algorithm using two gold-standard data sets.

**Conclusions:**

Our algorithm can identify protein complexes that are significantly enriched in the gold-standard data sets. Furthermore, our method surpasses three competing methods: MCL, ClusterOne, and MCODE in terms of the quality of the predicted complexes. The source code and accompanying examples are freely available at http://faculty.kfupm.edu.sa/ics/eramadan/GACluster.zip.

## Background

Protein–protein interaction networks are known to exhibit modular structure. A module in a protein interaction network could be a protein complex, an organelle, proteins involved in a functional pathway, etc [1]. Identifying the complexes within a protein interaction network is a challenging task due to two factors: First, interaction data from current high throughput methodologies have significantly high false positives and negatives. Second, a protein could belong to multiple complexes. We propose a protein complex discovery algorithm that uses genetic algorithms (GA) to identify complexes in protein interaction networks from yeast. Compared to earlier clustering algorithms proposed for this problem, our algorithm possesses several advantages that are enumerated below.

1. This approach recognizes that protein complexes are not cliques or near-cliques; the method is capable of identifying clustering with varying densities depending on the local density of edges in subnetworks (i.e., in dense regions of the network, it clusters dense subgraphs; and in sparse regions of the network, it clusters sparse subgraphs).
2. The approach is more robust and scalable. An example of this is that the clustering algorithm is capable of clustering large–size networks (such as the human protein interaction network), or it can cluster a large number of networks (hundreds of bacterial networks) without problems by ensuring that the many steps of the algorithm have costs that increase modestly with the number of nodes and edges in the network.
3. The algorithm can be tuned using parameters to obtain clusterings with a desired density and an average size of clusters.

## Related works

Three major graph clustering approaches have been employed to identify protein complexes.

The first approach searches for subgraphs with specified connectivities, called network motifs, and characterizes these as complexes or parts of them. A complete subgraph (clique) is one such candidate, but other network motifs on small numbers of vertices have been identified through exhaustive searching. Due to the time-complexities involved, this approach is restricted to searching for small subgraphs in large networks.

In the second, graph–growing approach, a cluster is grown around a seed vertex using graph search algorithms (greedy algorithms). These are local algorithms that begin with single, or several known nodes and then expand from there. The MCODE algorithm (Bader and Hogue [2]) starts with a single seed vertex, and adds more vertices based on a pre-computed set of weights. A vertex in the neighborhood of a cluster is added to it as long as its weight is close (within a threshold) to the weight of the seed vertex. Similarly, Bader [3] proposed the SEEDY algorithm, which progressively adds proteins to a seed protein to form complexes, based on a particular distance metric. Another software package called Complexpander by Asthana et al. [4] functions in this way to help identify protein complexes, including the seed proteins from a PPI network. However, our experience comparing this approach with the graph (global) clustering approach that we describe next shows that this approach is less *stable* than the latter (i.e., the clusters discovered depend strongly on the seed vertices chosen).

The third approach, the graph clustering approach, includes many variants. Algorithms in this category attempt to maximize or minimize certain cluster measures such as connection density, edge cut, or a novel distance metric between nodes in a cluster. In general, these are global algorithms that seek to optimize an objective function for the whole graph. One algorithm by Spirin and Mirny [5] employs the super-paramagnetic clustering (SPC), which is a technique based on a principle observed in physics to maximize the cluster density. Another algorithm by Przulji et al. [6] uses the concept of a minimal cut, which is a partition of the nodes of the network into two complementary sets such that the least number of edges cross from one set to the other. In their method, they perform recursive minimal cuts until they end up with densely connected subgraphs. Another method by King et al. [7] called restricted neighborhood search clustering (RNSC) begins by randomly assigning nodes to clusters, then reassigns nodes so as to minimize a cost function. Yet another such method by Enright et al. [8] uses a method called Markov clustering (MCL) to simulate the “flow” of the matrix. It does this by calculating increasing powers of the network’s adjacency matrix. With the increased powers, the areas of high flow become increasingly separated from those with little flow.

The methods described so far compute exclusive clusterings, i.e., they permit nodes to be members of at most one cluster. However, in biological systems many proteins and gene products participate in multiple functions [9]. Pereira-Leal et al. [10] used the MCL clustering algorithm in order to detect overlapping clusters. Their algorithm first turns a network with individual proteins as nodes, into a network with protein interactions as nodes (the line graph of the input graph). Then, the MCL algorithm is used to cluster the network of interactions. Finally, the algorithm re-converts the identified clusters from the interaction line graph back to the original graph with proteins as nodes. When the interaction network clusters are converted back to the original network, the same protein can appear in multiple clusters. Nepusz et al. [11] proposed the ClusterOne algorithm in order to detect overlapping clusters that is very similar to MCODE by starting from a single seed vertex. But the algorithm merges each pair of groups where the overlap score is above a specified threshold. Finally, it removes all clusters of a size less than three vertices or whose density is below a given threshold. Ramadan et al. [12] used the spectral clustering algorithm in order to detect overlapping clusters. Their algorithm first find all possible exclusive clusters using the spectral clustering method. Upon identifying all of exclusive clusters, it defines bridges (nodes that are significantly connected to two or more clusters) by examining the boundary nodes in the exclusive clusters (nodes that are joined to other nodes outside the cluster). This gives highly connected clusters, but still permits overlapping clusters, as nodes in one cluster may be involved in another cluster.

Another overlapping clustering algorithm is the PROCOMOSS algorithm proposed by Anirban et al. [13]. The PROCOMOSS algorithm detect overlapping clusters using the genetic algorithm technique. They rely on the properties captured in the graph modeling the PPI network, and they also utilize the GO terms to consider the biological properties of the proteins. Their approach can be described as follows: First, encode the chromosome as a vector of integer numbers representing the indices of the proteins in the proteins set. Then, initialize the population based on applying k-means clustering on both dimensions of the adjacency matrix *A* of a graph modeling PPI network. Next, calculate the fitness values of each individual of the population using two objective functions. Finally, select parents by adopting the same method used in NSGA-II [14] and mutate the selected chromosome as follows: select a random node and then either remove that node or add its neighbors to the selected chromosome with the same probability. The main drawback of this algorithm is that the predicted clusters cover a small percentage of the network.

## Methods

### Genetic algorithm

Genetic algorithm (GA) is a bio-inspired meta–heuristic algorithm that generally founded on the theory of evolution [15]. GA searches for optimal solutions by sampling the search space at random and creating a population of candidate solutions. GA uses genetic operators (e.g., mutation and crossover) to evolve into a population of new generations that is hopefully fitter according to a given objective (fitness) function. Survival of an individual to the next population is normally based on its fitness; that is survival of the fittest. However, the survival strategy normally does not preclude the survival of the less fit. Using GA to solve a given problem requires the following problem-dependent design: genetic representation of the problem solutions, the fitness function, candidate selection methods, and genetic operators (,e.g., crossover and mutation). The basic steps of GA are the following [16] :

- Create an initial population of candidate solutions.
- LOOP until any/all the candidate solutions become solution(s).
  1. Compute the fitness values of each of these candidates.
  2. Select candidates based on their fitness values.
  3. Create offspring from selected candidates using genetic operators
  4. Mutate each of these offspring using genetic operators.

### Spectral clustering

The graph clustering problem is that of finding the highly connected subgraphs (HCS) within the graph. The spectral clustering algorithm works by finding the minimum cut between two HCS subgraphs (clusters). The cut is the number of edges between the two distinct clusters. Finding the minimum cut is solved by the eigenvector *x*^∗^ corresponding to the smallest positive eigenvalue of the generalized eigen problem

$$Q x = \lambda D x, $$

where Q and D are the Laplacian matrix and the diagonal matrix of the graph, respectively. We consider the graph initially as one cluster, and proceed to obtain two clusters from it. We choose the size of the two clusters by applying the *k*-means clustering algorithm on *x*^∗^ with *k*=2 to choose the value of the eigenvector component that makes the objective function value is as small as possible. By a recursive application of this procedure, we obtain a clustering of the entire network. The number, size, and density of the clusters is determined by the network topology and the threshold value of the objective function used to determine if a cluster will be split again, and are not pre-specified [17].

We apply a spectral clustering method to identify initial subnetworks and clusters in the Collins protein interaction network.

### Objective functions

In this paper, we use the following three objective functions [18] to evaluate the quality of possible cluster structures. We compare the clustering achieved using these objective functions to the one achieved by our proposed objective function discussed later. We also compare clustering of all four objective functions to two gold standards.

- Min-Max-cut:
  $$ \text{JM}_{cut}(V_{1},V_{2})=\frac{W_{12}}{W_{11}} + \frac{W_{12}}{W_{22}}. $$
- Ratio cut:
  $$ \text{JR}_{cut}(V_{1},V_{2})=\frac{W_{12}}{|V_{1}|} + \frac{W_{12}}{|V_{2}|}. $$
- Normalized cut:
  $$ \text{JN}_{cut}(V_{1},V_{2})=\frac{W_{12}}{d_{1}} + \frac{W_{12}}{d_{2}}, $$
  where $ d_{k}=\sum _{i \in V_{k}} d_{i} $ the degree of each vertex belongs to *V*_*k*_ and *k*={1,2} and
  $$W_{il} \equiv W(V_{i}, V_{l}) = \sum\limits_{j \in V_{i}, k \in V_{l}, (j,k) \in E} w_{jk},$$
  where *i,l*=1,2 and *w*_*jk*_ is the weight on edge *jk*.

### Clustering algorithm

**Fig. 1:**
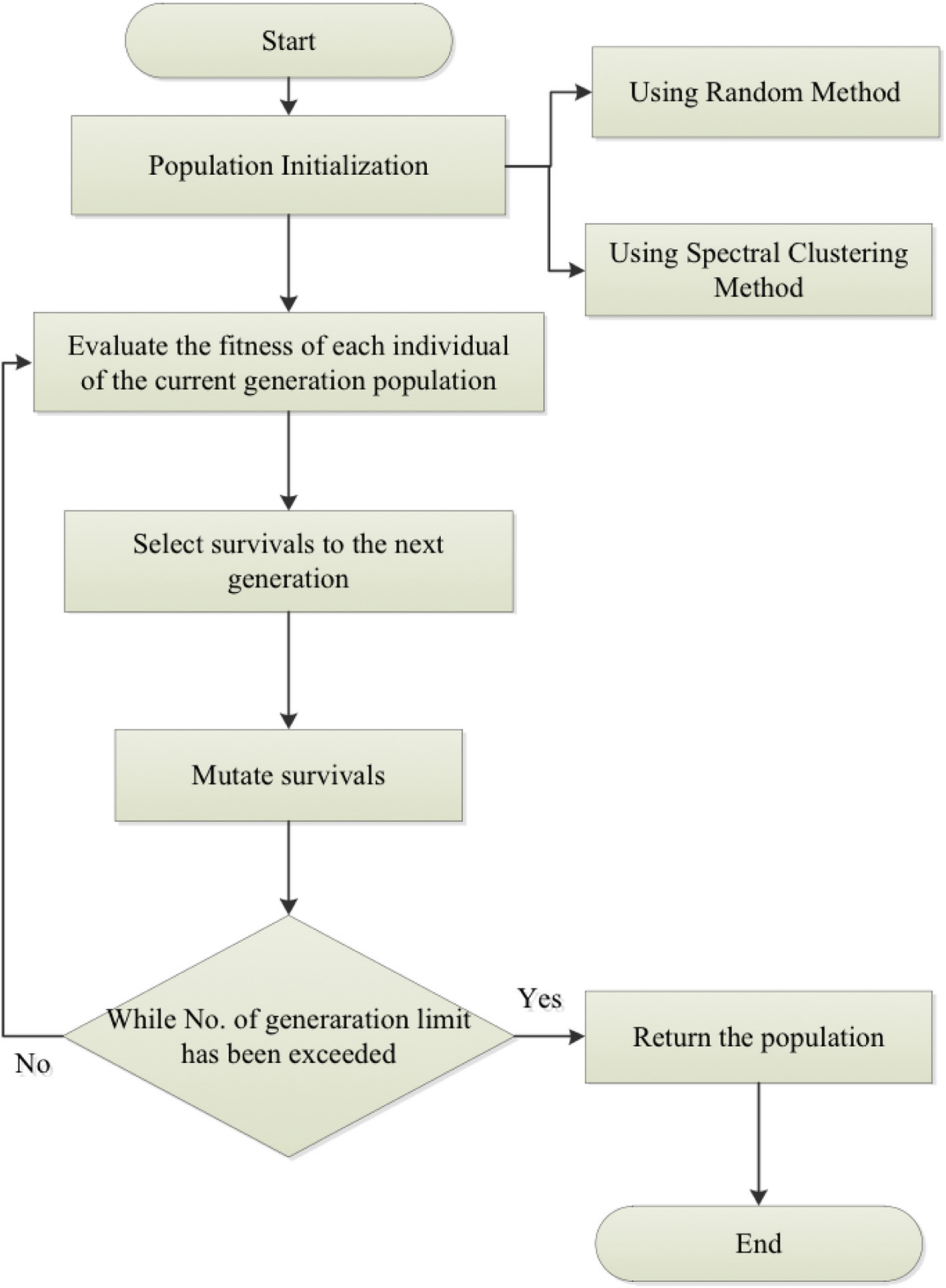
Clustering algorithm flowchart



In this section, we present a new overlapping clustering algorithm to help facilitate the different demands and purposes of cluster analysis. The structure of the new overlapping clustering algorithm, Algorithm 1, is shown in Fig. 1. Algorithm 1 employs GA for clustering the PPI network. Starting with an initial population of individuals (set of clusterings), the algorithm generates a new set of individuals using genetic operators (selection and mutation). The goal is to get individuals to converge to solutions (clusterings) of maximum fitness according to the objective function.

### Representation and initialization

**Fig. 2:**
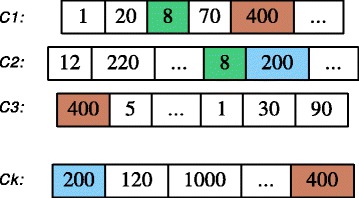
Population initialization

We represent each individual (possible solution for the problem) as *k* lists {*c*_1_,*c*_2_,*c*_3_,...,*c*_*k*_}, where *k* is the number of clusters. Each list can store integer numbers in the range {1,2,...,*N*}, where *N* is the size of the data set, as illustrated in Fig. 2. The element *j* of a list is a node’s index of the graph *G* modeling the PPI network. It is possible that some elements of different lists can hold the same value *j*, which means that a protein with index *j* can exist in more than one cluster; this is in case of overlapping clustering.

The population is composed of a number (population size) of individuals, or possible clusterings. We use two different methods to initialize the population. The first approach generates *m* random individuals, where *m* is the size of the population, as follows: for each individual consisting of *k* lists, assigning an integer value *j* in the range {1,2,...,*N*}, where *N* is the size of the data set for each element randomly. For example, as illustrated in Fig. 2, the node with index 70 is assigned to the cluster *c*_1_, while the node with index 8 is assigned to two clusters *c*_1_ and *c*_2_. Such a method should take into account the variety among the individuals of the population, which is supposed to be rather high.

In the second approach, we use the resulting clusterings of the spectral clustering algorithm [18] to create the initial population.

### Density–based objective function

The objective function aims to calculate the fitness values for each individual of the population to indicate how well each individual is suited to be the solution of a given problem. In our case, the fitness value of an individual reflects the intra–cohesion of each cluster proposed by the individual, as well as the inter–cluster coupling of those clusters. The goal is to maximize intra-cohesion and minimize inter-coupling. We represent intra-cohesion and inter-coupling by the number of edges within and across clusters, respectively. We compute the fitness of an individual as follows:

$$\text{JD}_{cut}(C_{1},..., C_{k})=\sum_{k} \frac{W_{kk}}{A_{k} + W_{ki}}, $$

where *W*_*kk*_ is the number of edges in a cluster *C*_*k*_, *W*_*ki*_ is the number of edges that has one endpoint in *C*_*k*_, and *A*_*k*_ is the maximum possible number of edges in the cluster *C*_*k*_.

### Genetic operators

The most common operations used in genetic algorithms are selection, crossover, and mutation. We exclude the crossover operation because it creates too many explorations that disturb the potentially good solutions. Regarding the parent–selection process, it is defined as the process of selecting individuals from the current population to create offspring for the next generation. This process aims to emphasize that the individuals with high fitness values are chosen in hopes that their offspring will have higher fitness as well. There are many ways to select parents, or individuals, from the current population for reproduction. Algorithm 2 illustrates in detail the parent–selection process.



**Fig. 3:**
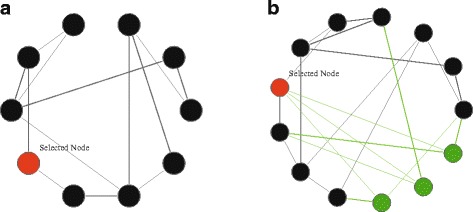
The mutation operation. **a** shows the selected node of the cluster c. **b** shows the cluster c after the mutation operator



The mutation operation is defined as performing some changes in the values of a specific chromosome, or individual. Consequently, the GA may reach to a better solution with the obtained individuals. We adapt the mutation operator used in [13] and modify it in such a case to be suited to, and more efficient for, our problem. This operation can be described as follows: after selecting an individual to be mutated, its nodes are either moved from one cluster to another, or some nodes of the network are added to the selected individual, as shown in Fig. 3. Figure 3a shows the selected node of the cluster and Fig. 3b illustrates the cluster after adding the selected node’s neighbors from the network. Algorithm 3 illustrates in detail the mutation process.

### Quality assessment

We consider an approach for quality assessment that finds statistically significant matches between discovered clusters and the reference data such as *precision* (*P*), *recall* (*R*), and *F*−*measure* (the harmonic mean of precision and recall) [19]. This approach measures the level of correspondence between discovered clusters and the reference data set by computing statistically significant matches between the two collections using hyper-geometric *p*-value, and used these matches to evaluate the precision and recall of the suggested clustering solution as follows. Let $\mathcal {C}$ be the initial set of discovered clusters, and let $\hat {\mathcal {C}} \subseteq \mathcal {C}$ be the subset of clusters that had a significant match based on hyper-geometric *p*-value.

Here, *p*-value is used to determine whether a discovered cluster is annotated by certain terms from the reference data set at a frequency greater than that would be expected by chance. It is calculated according to the following hypergeometric distribution:

$$p-value=1- \sum_{i=0}^{k-1}\frac{\left({\begin{array}{cc} M \\ i \end{array} } \right) \left({\begin{array}{cc} N-M \\ n-i \end{array} } \right)}{\left({\begin{array}{cc} N \\ i \end{array} } \right)}, $$

where *N* is the total number of proteins, *M* is size of a list of proteins *G* marked to the reference term of interest (protein complex), *k* is the number of proteins in a discovered cluster *C*, and *i* is the number of proteins shared between *C* and *G*.

For each predicted cluster *C*, let true positive (TP) be the set of proteins shared between the cluster *C* and a reference protein complex *G*, while false positive (FP) is defined as the set of proteins that exist only in the cluster *C*, and true negative (TN) is defined as the proteins that are members of the reference complex *G* but not found in the cluster *C*. Hence, P, R, and F-measure are calculated according to the following equations:

$$\begin{array}{ccc} \mathrm{P} & = & \frac{\text{TP}}{\text{TP} \cup \text{FP}}, \\ &&\\ \mathrm{R} & = & \frac{\text{TP}}{\text{TP} \cup \text{TN}}, \\ &&\\ \text{F-measure}&= &2 \times \frac{\mathrm{P} \times \mathrm{R}}{\mathrm{P} + \mathrm{R}}. \end{array} $$

## Results and discussion

### Data source

We study the protein interaction network from the yeast organism since there are abundant high-confidence data sets for its protein interaction network. In our experiment, we applied our clustering algorithm on the Collins protein interaction network extracted from the BioGrid data set [20]. This network has 8,319 interactions among 1,004 proteins. It has an average degree (16.57), where the degree of a node in a network is the number of links connected to the node; the density of this network is 0.016 (density is the ratio between the total number of connections and the potential connections that can exist in the network).

High–quality data collections are needed as gold standards to validate clustering approaches. We assess the coherence of the discovered clusters based on the Gene Ontology (GO) [21]. We have used the cellular component ontology from GO as the primary gold standard to compare the clusters obtained from the interactions data. We used the cellular components ontology in the GO since it includes more proteins in the protein interactions network than the other ontologies. We have also used collections of protein complexes in the yeast that have been culled from the literature and cataloged in the MIPS yeast genome database [22], as well as a hand-curated reference complex set called CYC2008 [23].

### Clustering comparisons

We compare the performance of our algorithm (with different objective functions and initializations) with some of the methods mentioned in the related works section, which were commonly utilized for extracting complexes from protein interaction networks. We report the performance measures that were mentioned earlier. Table 1 presents a comparison of the performance of our algorithm (when the population is initialized using initial clusters through spectral clustering or initial random clusters) with other existing methods for clustering for the Collins data set. We used CYC2008 complexes and MIPS complexes as the reference data sets in order to compute the performance measures.

**Table 1:** Comparison of clustering algorithms on the Collins network. The populations in our method are initialized using spectral and random clusterings

Method	#Cls	CYC2008			MIPS			Discard
		R	P	F-measure	R	P	F-measure	
MCODE	54	0.66	0.59	0.63	0.27	0.48	0.35	40 %
MCL	75	0.65	0.45	0.54	0.27	0.34	0.30	19 %
ClusterOne	114	0.55	0.43	0.49	0.20	0.34	0.25	18 %
Our method using								
spectral initialization								
1) Density cut	162	0.74	0.60	0.66	0.32	0.45	0.37	14 %
2) Maxmin cut	180	0.71	0.47	0.60	0.38	0.40	0.39	15 %
3) Normalized cut	193	0.67	0.50	0.57	0.39	0.37	0.37	20 %
4) Ratio cut	161	0.73	0.38	0.50	0.39	0.33	0.36	17 %
Our method using								
random initialization								
5) Density cut	164	0.72	0.54	0.62	0.30	0.41	0.35	18 %
6) Maxmin cut	162	0.71	0.45	0.56	0.40	0.35	0.38	17 %
7) Normalized cut	138	0.66	0.57	0.61	0.36	0.44	0.41	19 %
8) Ratio cut	154	0.61	0.55	0.58	0.34	0.43	0.38	18 %

A study by Brohee and van Helden [24] that compared these algorithms (among others) showed that the MCODE and MCL algorithms, in particular, were very effective in identifying protein complexes from protein interaction networks. We investigated the performance of our method when compared to these two algorithms. In addition, we also investigated the performance of one of the recent algorithms for clustering (the ClusterOne algorithm). In short, we used the MCODE, MCL, and ClusterOne algorithms to extract clusters from the yeast Collins network.

Clearly, our clustering algorithm (Algorithm 1), which was based on initial spectral clustering and used density cut as an objective function (version 1), has the lowest discard ratio (14 *%*) over all the other approaches; a low value of discard ratio indicates that a high proportion of the proteins in the considered protein network are clustered. On the other hand, the MCODE algorithm has the highest discard ratio (40 *%*) because it searches for high–density clusters only. Also clustering algorithm (version 1) yields a high precision value with CYC2008, and also a high recall value (most complexes formed by the proteins under study overlap well with the computed cluster from the protein network). MCODE has a similar results, but with one major drawback, which is that not all the proteins in the network are clustered, as illustrated by the high discard value. It can be seen that our clustering algorithm outperforms the MCODE algorithm by a significant margin in terms of discard and recall values. In addition, our algorithm with different objective functions and initializations (versions 1–8) usually discover more clusters, while MCODE predicts fewer clusters; and the other approaches, MCL and ClusterOne, predict fewer clusters than our method and more clusters than MCODE, as illustrated in Table 1.

In comparison with the MCL and ClusterOne algorithms, our algorithms exhibit better correspondence with the complexes catalog within CYC2008 data set, and has higher recall and precision levels than those attained by the MCL and ClusterOne.

### Clustering quality

We assess the biological significance of the clusters in the Collins network by comparing them with components in the Gene Ontology. We use the GO term finder [25] to get the most significant GO-terms, GO-id, and *p*-values for a list of proteins (predicted complex). Table 2 tabulates some of the clusters of the Collins network that have a significant *p*−value. Each cluster is listed by its ID used in this study, and the number of proteins in it. Also tabulated is the number of cluster proteins in a GO component that has the highest overlap with it. This number is expressed as a percentage (N%). These percentages are 100 for most clusters, showing that these clusters in the network overlap well with the corresponding GO components. Proteins in a GO component are not found in the cluster, mostly when the proteins are not present in the Collins network. Table 2 clearly shows that genetic algorithm–based methods are capable of identifying the protein complexes.

**Table 2:** A few of the clusters in the Collins network with the lowest *p*-values with GO components

#	Size	GO-ID	GO-Term	*p*-value	N%
1	17	GO:0030880	RNA polymerase complex	3.30986E-39	100.0 %
2	8	GO:0044428	Nuclear part	3.70274E-05	100.0 %
3	7	GO:0030126	COPI vesicle coat	1.37069E-21	100.0 %
4	14	GO:0044428	Nuclear part	7.23152E-10	100.0 %
5	27	GO:0005739	Mitochondrion	9.82318E-22	100.0 %
7	18	GO:0000502	Proteasome complex	1.76807E-40	100.0 %
8	12	GO:0005634	Nucleus	3.90352E-06	100.0 %
9	7	GO:0030008	TRAPP complex	1.02802E-20	100.0 %
11	21	GO:0005634	Nucleus	2.04087E-10	100.0 %
12	10	GO:0044425	Membrane part	4.18992E-10	100.0 %
13	5	GO:0035097	Histone methyl–transferase complex	1.31389E-11	100.0 %
14	5	GO:0030126	COPI vesicle coat	1.18247E-14	100.0 %
15	9	GO:0016585	Chromatin remodeling complex	2.37606E-17	100.0 %
16	15	GO:0000502	Proteasome complex	2.20275E-33	100.0 %
17	13	GO:0043189	Histone acetyl–transferase complex	1.21627E-39	100.0 %
20	12	GO:0016514	SWI/SNF complex	4.98150E-37	100.0 %
21	60	GO:0005634	Nucleus	2.15384E-32	100.0 %
22	81	GO:0043227	Membrane-bound organelle	4.87516E-23	100.0 %
24	63	GO:0044464	Cell part	3.42642E-05	98.4 %
23	4	GO:0031011	INO80 complex	4.13601E-07	75.0 %

The GO component that has the lowest *p*-value with these clusters is listed, the number of proteins in the cluster that overlap with the GO component are listed as percentages of the number of proteins in the cluster (N%). *p*-values defined in the text are also shown

## Conclusion

In this paper, we proposed a robust approach for identifying protein complexes in PPI networks. The approach takes advantage of GA to help address the complex and heterogeneous nature of protein networks clusterings. We designed a new objective function to allow, overall, for the maximizing of intra-cluster cohesion, and the minimizing of inter-cluster coupling. Experimental results have shown that our objective function performs better than other objective functions proposed in the literature to identify overlapping clusters in PPI networks. In general, our clustering approach is found to be more accurate and consistent than existing methods (i.e., MCL, ClusterOne, and MCODE) when compared with two reference sets: MIPS and CYC2008, using the Collins network.

In conclusion, our approach outperformed competing approaches and is capable of effectively detecting both dense and sparsely connected biologically relevant protein complexes with fewer discards.
